# To have and to hold: looking vs. touching in the study of categorization

**DOI:** 10.3389/fpsyg.2015.00178

**Published:** 2015-02-18

**Authors:** Lynn K. Perry

**Affiliations:** Department of Psychology, University of Wisconsin-MadisonMadison, WI, USA

**Keywords:** categorization, novel noun generalization, manual exploration, context, looking

In order to make sense of the “blooming, buzzing, confusion” of a world where no two objects or events are ever exactly identical, infants form categories of perceptually distinct items that can be treated equivalently. The study of categorization often arises from one of two motivations: (1) to examine the mechanisms by which infants learn to treat distinct objects/events as equivalent (e.g., how an infant comes to name two different animals dog) and (2) to examine the current state of an infant's knowledge (e.g., does an infant *have* a category of dog?). As an illustration, consider Quinn et al.'s (1993) study. After habituating to different dog pictures, 3- and 4-month-olds do not dishabituate to cats. However, after habituating to cats, they *do* dishabituate to dogs. If we were examining the current state of knowledge, what should we conclude? Unless we want to suggest these infants are participants in Schrödinger's thought experiment (1935)—they both have and do not have a cat category—we cannot say anything conclusive. Instead, the results reveal something important about the mechanisms of categorization. Quinn et al. (1993) showed variability between category members affects the exclusivity of the categories infants form. This example demonstrates that studying whether infants *have* a category misses the crucial point that categories are neither things that people have in their heads nor that exist in the world; but rather categorization is a process (see also Oakes and Madole, 2000; Samuelson and Smith, 2000; Samuelson et al., 2007).

In addition to these two main motivations, the study of categorization typically utilizes one of two methodologies: (1) *looking measures*, e.g., habituation and preferential looking, and (2) *touching/reaching measures*, e.g., sequential touching or manual forced choice measures. What I will argue in this paper is that looking measures (1) too readily lend themselves to interpretations of infants having a category or not, and (2) miss the importance the body and physical context have on development.

## A temptation of looking

Too often the focus of looking measures is the outcome—do infants look longer at one stimulus—without regard for how past experiences and the present context influence infants' looking (but see, Perone and Spencer, 2013). Interpretations that infants do or do not *have* a category have proven to be particularly tempting in studies examining infants' categorization of physical events (see also Haith, 1998). For example, evidence that 3 to 4-month-old infants habituated to a ball rolling behind an occluder dishabituate to an impossible event—a ball rolling behind an occluder and not stopping at a barrier—has been interpreted as evidence for infants having knowledge of physical laws of solidity (Spelke et al., 1992). However, in experiments employing reaching measures, when a ball rolls behind a panel of doors and stops at a barrier to the right of one of the doors, older children fail to select the correct door, instead perseveratively reaching for the same door regardless of the barrier location (Berthier et al., 2000). This failure makes it difficult to say whether infants *have* knowledge of physical laws.

However, failure can tell us something more interesting about development (Keen, 2003; Perry et al., 2008, 2009). For example, Perry et al. (2009) demonstrated changes in children's ability to align their body and movements with relevant spatial reference frames support search accuracy. When 2-year-olds underwent training with the barrier in a fixed position while the ramp moved, such that the correct door was aligned with midline, they correctly located the object during the standard testing procedure. The context of children's environment—where their bodies are relative to objects and events in the world—influences where they attend and subsequently remember. Such findings demonstrate that the context of learning is critical to understanding developmental change—findings that would not have been made through looking measures. This realization can be applied to the study of categorization.

The widespread use of habituation beginning in the 1980s means that much of our knowledge about infants' categorization comes from their visual exploration of (often) static images (or occasionally visual stimuli in the context of auditory stimuli). Looking measures offer advantages over touching/reaching measures. They can be used with young infants with poor motor control; and computer displays allow increased precision in stimuli construction and presentation. The ability to conduct experiments with a variety of age groups and with high precision has benefited the study of many developmental phenomena, including categorization.

However, looking methods can only tell us about categorization of visual or auditory stimuli. A growing body of evidence suggests that word learning and categorization are often closely tied to other perceptual and sensori-motor aspects of the learning context (see e.g., Samuelson et al., 2011; Perry et al., 2014). Additionally, the particular task used to measure categorization matters. For example, researchers investigating basic and superordinate-level categorization discovered infants generally form broader categories (corresponding to adult-defined superordinate-level categories such as animal and vehicle) during touching tasks (see e.g., Mandler and Bauer, 1988; Mandler et al., 1991; Mandler and McDonough, 1993; and see Mandler, 2000 for review) and narrower categories (corresponding to adult-defined basic-level categories such as horse and zebra) during looking tasks (see e.g., Roberts and Cuff, 1989; Behl-Chadha, 1996; and see Mandler, 2000 for review). Thus, relying *solely* on looking measures therefore provides an incomplete picture of categorization. Next, I discuss evidence from one particular type of categorization—novel noun generalization—that illustrates an essential role for manual actions in learning.

## Novel noun generalization: a test case for touching

Children develop word-learning biases that help them generalize novel names to novel objects. By 2-years-old, children typically generalize names of novel solid objects by similarity in shape (shape bias). By 4-years-old, they typically generalize names of novel non-solid substances by material (material bias). Researchers debate what generalization by shape or material similarity says about the role of perceptual and conceptual information in children's category learning (see Samuelson and Bloom, 2008). The attentional learning account proposes biases emerge out of associations between perceptual and linguistic regularities (e.g., Samuelson and Smith, 1999; Yoshida and Smith, 2005; Perry and Samuelson, 2011). On the other hand, knowledge-based accounts argue biases are driven not by lower-level regularities but higher-level knowledge about, e.g., functions and core properties (Hammer and Diesendruck, 2005; Kemler Nelson and 11 Swarthmore College Students, 1995). Importantly, both accounts recognize the effects of context in categorization. For example, children's tendency to demonstrate the shape bias (Hammer and Diesendruck, 2005) and the material bias (Samuelson and Horst, 2007) varies with the specific stimuli and task used. Context-dependency is not a problem of a task that needs to be removed, however, but a fact of categorization. Categorization is not static, but emerges as prior knowledge is brought to bear in a given context (see e.g., Samuelson et al., 2007). Converging evidence supporting this view also suggests an important role for manual action in categorization.

Smith (2005) found that the actions children performed on an object influenced how they generalized its name. When 2-year-olds were told a novel object was a “wug” and taught to move it up and down, they generalized the name to an object elongated on the vertical axis rather than one elongated on the horizontal axis. Critically, the child had perform the actions, as children in a condition who watched the experimenter perform the action did not systematically select objects elongated consistent with the movement (Smith, 2005). By examining children's manual interactions with real, physical objects, these results demonstrate that the actions children perform with objects determine what information they use for categorization.

Similarly, Perry et al. (2014) found that children's understanding of non-solid substances (e.g., pudding) is influenced by context-dependent actions. At home, toddlers primarily learn about non-solids during mealtimes, when they sit in a highchair and can touch and eat non-solid foods. When 16-months-olds sat in a highchair in the laboratory, they were more likely to messily touch novel substances than those seated at a table. The messier children were, the more likely they were to generalize names of novel non-solid substances by material. The highchair is a cue to a context-dependent action pattern supporting attention to material. Critically, children who did not touch the substances failed to generalize novel names by material, suggesting looking is not enough. We would miss important discoveries if we only used looking measures to study children's novel noun generalization. What the touching method afforded was the ability to see *how* children learn about material.

Together these studies reveal context is not a factor to eliminate from experiments, but instead an inseparable part of categorization. Responses in these studies were measured via touching (which object/substance did the child hand to the experimenter). Nevertheless, contrasting touching (or amount of touching in Perry et al.'s study) with looking *during learning* revealed children's manual actions determine the information they use to generalize. Moreover, by showing how critical actions are to learning, these studies provide evidence for the usefulness of employing touching measures in studying categorization.

## Why touching matters

Mandler (2000) proposed an important difference between looking- and touching-based tasks: infants are more interested in and more actively explore objects than static images. Infants develop and explore within a world full of rich multimodal information. Indeed, even adults' perception of familiar objects is influenced by manual exploration (Lederman and Klatzky, 1990; Yee et al., 2013). Embodied cognition theorists posit exploration and the multimodal nature of experiences are critical to development (Thelen and Smith, 1994; Smith and Gasser, 2005). Redundant information across modalities (e.g., visual, haptic) facilitates learning of more abstract information (see Bahrick et al., 2004).

Cross-modal redundancy is especially useful for understanding shape and material. As an infant explores a toy, haptic information is integrated with visual information. Over development, exploration changes representation of that toy's shape, and objects' shapes in general (e.g., Soska et al., 2010). The ability to recognize an object by its shape, is a critical precursor to developing a shape bias (Yee et al., 2012). As infants explore non-solids, there is similarly redundant sensory information. Nonsolid substances are particularly difficult to recognize by vision alone (cf. Adelson, 2001)—e.g., visual similarity between milk and glue is high, while haptic similarity is low. Over development, exploration increases infants' ability to learn subtle visual cues associated with different materials—critical for categorization of non-solids (see also, Perry et al., 2014).

Given how important multimodal interactions are to development, we as a field cannot rely on looking measures at the expense of what can be learned studying categorization in the context of real objects. Looking measures focusing only on learning *outcomes* cannot reveal the processes driving categorization. Nevertheless, looking measures have a place in the study of categorization. As critical as touching is to infants' development, not every question is a touching question. Studies examining where infants look during learning have provided important insights into what infants glean from a given context and subsequent effects on object perception (Johnson et al., 2004) and categorization (Best et al., 2013). Studying looking as an exploratory process and not just an index of cognition—whether using eye-tracking or traditional habituation—will be a fruitful complement to studies of manual exploration.

## Conclusions

Categorization is a critical process by which we make sense of the world. Importantly, when children learn about individual categories, they are also learning higher-order regularities between categories, changing the way they learn categories in the future (e.g., Perry et al., 2010). Thus, studying children's categorization is valuable in understanding how knowledge and learning *change* over time. Given that knowledge is dynamically constructed with information from multiple senses, inseparable from context, and children are developing within a world where they are engaging these senses, we miss something important if we *only* study categorization using looking measures. As a field, we cannot ignore the richness of children's everyday actions and experiences; therefore, we must engage a variety of methodologies to understand development and the categorization process.

### Conflict of interest statement

The author declares that the research was conducted in the absence of any commercial or financial relationships that could be construed as a potential conflict of interest.
